# Further development of crew resource management training: Needs assessment by means of teamwork-context analysis in anesthesia and intensive care teams

**DOI:** 10.1007/s00101-022-01170-3

**Published:** 2022-07-08

**Authors:** Hendrik Eismann, Georg Breuer, Markus Flentje

**Affiliations:** 1 Department of Anaesthesiology and Intensive Care Medicine, Hannover Medical School, Carl-Neuberg-Straße 1, 30625 Hannover, Germany; 2 REGIOMED Kliniken, Ketschendorfer Straße 33, 96450 Coburg, Germany

**Keywords:** Teamwork context, Collective orientation, High responsibility teams, Simulation-based training, Teamarbeit, Kollektive Orientierung, High-Responsibility-Teams, Simulationstraining

## Abstract

**Background:**

Teams in anesthesia and intensive care work as high responsibility teams (HRT). Success in this environment partly depends on the use of nontechnical skills which can be learned through simulation-based training. A teamwork context analysis could help to identify training requirements for crew resource management training.

**Material and methods:**

We used a multicentric observational cross-sectional study design utilizing survey methodology to evaluate the teamwork context of different work environments, using the 62-item TAKAI inventory. We surveyed anesthesia and intensive care staff from nine hospitals in Germany which provide varying levels of care.

**Results:**

In total, 128 people (44.5% male, 53.9% female) from 9 German hospitals participated in the study. The topics “interconnectedness: departments”, “interconnectedness: information flow”, “dynamics”, “polytely”, “velocity of the team’s movement”, “velocity of system changes”, “hierarchy” and “hierarchy: leadership”, “shared task mental model”, “shared team mental model” and all aspects of the scale “adaptive behaviors” were identified as focal aspects to be implemented into Crew-Resource-Management (CRM) training for the evaluated work environments.

**Conclusion:**

The TAKAI scales meet quality criteria (Cronbach’s alpha > 0.6) and are appropriate for use in the analysis of the teamwork environment. The results indicate many similarities between the work contexts surveyed but also slight differences. TAKAI can be an additional method to design an appropriate simulation training program for HRT in anesthesia and intensive care medicine as there does not seem to be a one-size-fits-all simulation concept. For a special focus on the needs of a work context, the easy to perform TAKAI analysis in the needs analysis step is worthwhile.

**Supplementary Information:**

The online version of this article (10.1007/s00101-022-01170-3) contains further illustrations, the questionnaires and a table of the demographic data of the participants.

## Abstract (de)

**Hintergrund:**

Teams in der Anästhesie und Intensivmedizin arbeiten unter den Bedingungen von High-Responsibility-Teams. Erfolgsfaktoren in diesem Umfeld sind nichttechnische Fähigkeiten, die in simulationsbasierten Trainings trainiert werden können. Eine *Team-Arbeit-Kontext-Analyse* könnte helfen, den Trainingsbedarf für Crew-Resource-Management-Trainings zu identifizieren.

**Material und Methoden:**

In einer multizentrische Beobachtungsstudie wurde der Team-Arbeit-Kontext verschiedener Arbeitsumgebungen mit Hilfe des *Team-Arbeit-Kontext-Analyse Inventar* untersucht. Es wurde Personal der Anästhesie und Intensivmedizin aus neun Krankenhäusern in Deutschland befragt.

**Ergebnisse:**

Insgesamt nahmen 128 Personen (44,5 % männlich, 53,9 % weiblich) aus neun deutschen Krankenhäusern an der Studie teil. Die Themen „Vernetzung: Abteilungen“, „Vernetzung: Informationsfluss“, „Dynamik“, „Polytelie“, „Geschwindigkeit der Teambewegung“, „Geschwindigkeit der Systemveränderungen“, „Hierarchie“ und „Hierarchie: Führung“, „Gemeinsames mentales Aufgabenmodell“, „Gemeinsames mentales Teammodell“ und weitere wurden als Fokusaspekte identifiziert, die in CRM-Trainings implementiert werden sollten.

**Schlussfolgerung:**

Die TAKAI-Skalen können für die Analyse der Teamarbeitsumgebung verwendet werden. Die Ergebnisse zeigen viele Gemeinsamkeiten zwischen den untersuchten Arbeitskontexten, aber auch diskrete Unterschiede. TAKAI kann eine zusätzliche Methode sein, um ein geeignetes Simulationstraining für Teams aus der Anästhesie und Intensivmedizin aufzubauen, da es kein *One-size-fits-all* Simulationskonzept gibt. Für einen speziellen Fokus auf die Bedürfnisse eines Arbeitskontextes lohnt sich die einfache Durchführung der TAKAI-Analyse im Rahmen einer Bedarfsanalyse.

## Introduction and background

Teams working in the fields of anesthesiology and intensive care medicine are subject to the conditions of high responsibility teams (HRT) [19]. These working environments are characterized as complex and demanding. Errors, especially human factor-related errors, often have serious consequences including patient harm [2, 8, 23]. Factors for success and those which prevent accidents in HRT include non-technical skills such as task management, decision making, situational awareness, and teamwork [13, 27].

Additionally, improvements in technical and non-technical competencies by simulator-based training have been shown and regular attendance is requested by various European anesthesia societies [25, 32].

The Helsinki Declaration on Patient Safety in Anesthesiology calls for training on rare incidents as for example malignant hyperthermia (MH), local anesthetic systemic toxicity (LAST) or difficult airway management [25].

In Germany, training content, scenario design, and training sequence are highly variable as there are many simulation training facilities [1]; however, a differentiated analysis of the work environment is often missing when designing simulation scenarios. Such an analysis also seems necessary, considering that for effective training relevance for daily work, an open mind towards training is important [28]. Looking at changes in collective orientation (a concept to measure the willingness to work together in teams) we could identify multiple impacts of simulation training on different cohorts specific to the occupational groups [6, 8, 11].

For the training of teams in an obstetric environment, Draycott suggested evaluating the training by means of effectiveness as it is very cost-intensive [5]. Training focused on the specific needs of the targeted group of participants could help to increase the efficiency.

This is especially true in departments where high-risk, time-critical procedures are performed on critically ill patients as part of a multidisciplinary team as human factors are likely to play an important role [17].

Additionally, for medical curricula, a needs assessment is widely recommended. A consideration of the work environment might help to determine the individual needs [24].

A teamwork context analysis inventory (*Team-Arbeit-Kontext-Analyse Inventar,* TAKAI) enables searching for differences and commonalities in the different team work contexts “complexity”, “context criteria”, “shared mental model” and “adaptive behavior” and has been developed in cohorts from aviation and medicine, among others [19, 21].

The aim of this work was to create job profiles for the various roles in anesthesia and intensive care medicine and from this to derive a focused training design.

We hypothesized that a) the teamwork context differs in anesthesia and intensive care teams and b) content and training requirements for crew resource management can be derived from the teamwork context analysis.

## Study design and investigation methods

### Study design

This study has a multi-centric observational cross-sectional study design using a survey methodology. Participants were recruited from different hospitals in Germany.

This study was reviewed and approved by the Ethics Committee of the Medical School Hannover under the processing number 9445_BO_K_2020 (Chairperson: Professor S. Engeli).

### Setting and population

We sent the compiled questionnaire to executive personnel of nine hospitals providing varying levels of care in Germany. We asked them to distribute the questionnaire among anesthesia and intensive care staff. The German language version of the questionnaire is shown in *the* *online material. *Inclusion criteria for this study were a) participants must form part of the requested professional group (physician, nurse, anesthesia technician) and b) all participants were required to be active in the field of anesthesiology or intensive care medicine at the time of the study. Participation was voluntary and could be withdrawn at any time without giving reasons. We used SoSci Survey (SoSci Survey GmbH, Munich, Germany) as an online survey tool and sent a link attached to an information sheet about the background of TAKAI and the goals of the study. All participants had a 4-week time period to complete the survey. No reminder was sent.

### Questionnaire

We assembled a questionnaire using TAKAI with questions to assess the demographic data of the participants as well as two questions regarding the participants experience in the fields of “human factors” and “simulation as team training methodology”. We used the unchanged TAKAI questionnaire in the German language as developed and described by Hagemann et al. [21]. The scales and item groups are shown in Table 1.

**Table 1:** Scales and items of TAKAI (*Team-Arbeit-Kontext-Analyse Inventar*) with practical examples for the conceptualization of items and item groups [19]

Scale	Item or item group	Conceptualization
*Complexity*	Intransparency	Treatment information is incomplete or turns out to be incorrect during the course of treatment
	Interconnectedness: departments^a^	There are one-way or two-way interdepartmental dependencies. Cooperation may be necessary
	Interconnectedness: information flow^a^	Information is passed back and forth between different team partners
	Polytely^a^	In a situation, several goals must be pursued simultaneously, which may also contradict each other
	Dynamics^a^	If no action is taken, the situation will continue to evolve independently. The time for intervention and decision-making is limited
	Delayed feedback	Impact of processes on the overall system are unknown, yet need to be anticipated
*Context criteria*	Velocity of the teamʼs movement^a^	The team is not location-bound and moves while working. The speed of movement ranges from low (a pedestrian) to high (a fighter jet)
	System size (single item)	The number of people in a team varies from low (2 in the cockpit) to high (4–10 in the operating room (OR) or 10 in a technical team)
	Velocity of system changes^a^	The processes to which the team must react occur relatively quickly. Decisions need to be made with urgency
	Personal threat	At work, there is a risk that one’s life may be placed in danger during a critical situation
	Hierarchy^a^	Hierarchical structures and organizational processes significantly influence the behavior of team members
	Hierarchy: followership (single item)^a^	Instructions from superiors are questioned when necessary
	Hierarchy: leadership (single item)^a^	Comments from lower ranked staff will be considered
	Environmental factors	Environmental conditions such as heat, cold, storm, wetness and darkness influence the team and affect its performance
	Impairment of communication	During work, communication between team members is disrupted. Information is lost in the process
	Familiarity of the work environment	During work, the team does not go to places they have never been before. The work environment is familiar
*Shared mental model*	Shared task mental model^a^	A shared mental model of the environment and tasks (e.g. technologies, equipment, and interaction) exists among team members
	Shared team mental model^a^	There is a shared mental model of team members’ roles, interactions, and responsibilities. They have a shared understanding of the knowledge and skills, strengths and weaknesses, and attitudes of others on the team
*Adaptive behavior*	Information gathering (single item)v	Important information about the situation and the task at hand is gathered, applied to the work context, interpreted, and used to anticipate future problems
	Information interpretation (single item)^a^	The team gathers information and anticipates future conditions and potential problems
	Anticipation of conditions (single item)^a^	
	Task prioritization (single item)^a^	Tasks are sorted and processed according to their importance. Tasks can also be reprioritized
	Prioritization during recovery (single item)^a^	The team prioritizes work during rest periods to coordinate and work effectively
	Reprioritization (single item)^a^	Tasks are reprioritized by the team as needed
	Task management (single item)^a^	Tasks are distributed among team members. If necessary, the tasks can be redistributed, so that the workload remains the same for all team members
	Adaption of task management (single item)^a^	The team distributes the workload between the team members in order to create a balance and thus enhance the ability to act in time-critical and highly stressful situations

^a^Item should be prioritized when designing or developing crew resource management training or scenarios for anesthesia or critical care

The 62 items (14 item groups and 12 single items) were formulated unipolar, as frequencies of situations and behavior are assessed or approval must be given. Each level of the rating scale is marked with a number from 0 to 6 (7-point Likert scale) and named exactly, because exact naming improves the test quality of instruments as described by Hagemann et al. [3, 21]. The item “system size” represented numerically to reflect the number of people in each team. The term “team” was not defined in the questionnaire, the participants could define themselves what they consider as a team. The items 4, 7, 12, 18, 19, 33, 34, 36 and 43 were reverse calculated because the statements were phrased negatively. After recoding, we calculated the mean of the individual item group and single item.

### Statistical analysis

Demographic and survey data were analyzed in a descriptive manner. The internal consistencies of the scales were determined by Cronbach’s alpha. In order to test differences in the measured teamwork context, we conducted a Mann-Whitney U‑test. We assumed *p* < 0.05 as being statistically significant. The item “system size” was described as median and interquartile range (IQR). We performed Kruskal-Wallis tests to test differences between demographic data and the experience with simulation-based training. All calculations were made using SPSS Statistics 26 (IBM Corporation, Armonk, NY, USA).

## Results

### Demographic data

In total, 128 persons (44.5%:57 male, 53.9%:69 female 1.6%: 2 non-binary) from 9 German hospitals participated in the study (*see online material “Demographic Data”*). Of these 102 (79.8%) were from the working environment of anesthesia and 26 (20.3%) from intensive care medicine. The age distribution, levels of hospital care and work experience of the participants are shown in the *online material “Demographic Data*”. The total sample divides into five different professions (residents, consultants, nurses, specialized nurses and anesthesia technicians) and into two subgroups by their work environment (anesthesia, intensive care medicine) for further evaluation. An overview of the participant characteristics is shown in the *online material “Demographic Data”*.

### Experience with human factors, crew resource management and simulation-based training

We asked the participants to rate their experience in the areas “human factors”, “crew resource management” (CRM) and their experience with the training methodology “simulation”. The data is shown in the *online material “Additional Figures”.*

Of the participants 7% rated their experience with human factors and CRM with “no experience” (anesthesia: 6.9%, intensive care medicine: 7.7%), 4.7% rated their experience with “very much experience” (anesthesia 5.9%, intensive care medicine 0.0%). The largest percentage of respondents (46.9%) reported their experience as “moderate experience” or “some experience”.

Accordingly, 4.7% rated “no experience” with simulation-based training as team training (anesthesia: 4.9%, intensive care medicine: 3.8%), 6.3% rated with “very much experience” (anesthesia 5.9%, intensive care medicine 7.7%). The largest percentage of respondents reported their experience as “little experience” to “some experience” (67.2%).

We saw no difference in the level of care or gender in terms of staff experience with simulation-based training; however, we saw a significant difference in the age (*p* = 0.005), and work experience (*p* = 0.039) of the respondents. The staff with an age of 40–49 years and the staff with 10–15 years of work experience have significantly more experience in simulation than all other age categories.

### Reliability of the scales

For testing the reliability of the scales, we calculated Cronbach’s alpha. We found Cronbach’s alpha of 0.614 for the scale “complexity”, 0.621 for “context criteria”, 0.875 for “shared mental model” and 0.778 for “adaptive behavior”.

### Differences in the scales of TAKAI between the work environments

**Fig. 1:**
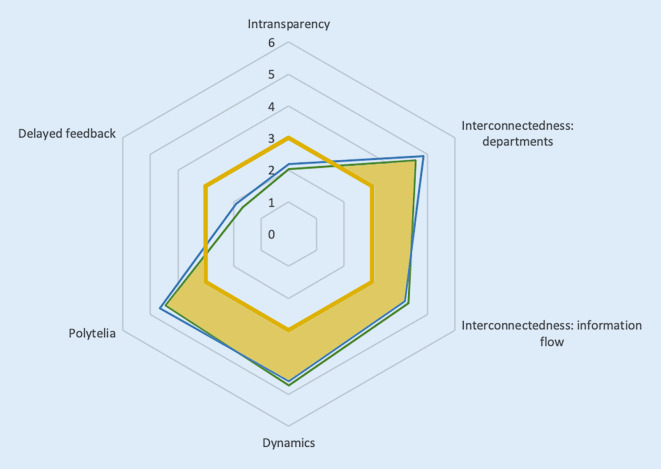
Radar chart of the scale “complexity” (*blue line*: anesthesia, *green line*: intensive care medicine). The aspects “interconnectedness: departments”, “interconnectedness: information flow”, “dynamics” and “polytely” should be implemented into CRM trainings. The *shaded areas* mark the items in which the measured data deviate from the cut-off values [19]

**Fig. 2:**
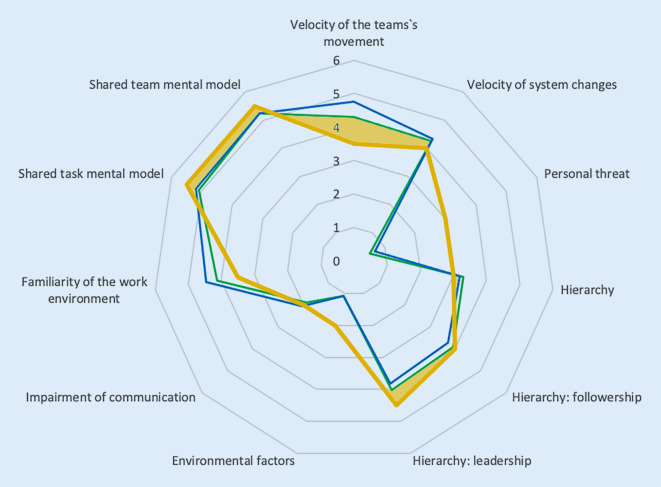
Radar chart of the scales “context criteria” and “shared mental model” (*blue line*: anesthesia, *green line*: intensive care medicine). The aspects “velocity of the teams’ movement”, “velocity of system changes”, “hierarchy” and “hierarchy: leadership”, “shared task mental model” and “shared team mental model” should be implemented into CRM trainings. The *shaded areas* mark the items in which the measured data deviate from the cut-off values [19]

**Fig. 3:**
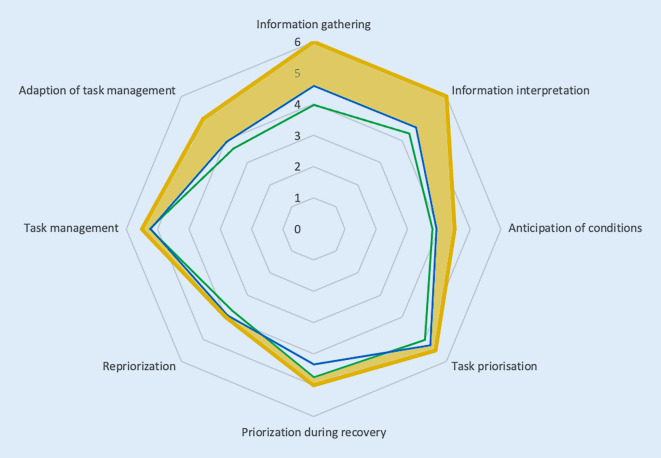
Radar chart of the scale “adaptive behavior” (*blue line*: anesthesia, *green line*: intensive care medicine). All aspects should be implemented into CRM trainings. The *shaded areas* mark the items in which the measured data deviate from the cut-off values [19]

The results of the TAKAI are shown as “job profiles” in Figs. 1, 2 and 3. The item group “familiarity of the work environment” showed a significant difference between the analyzed work environments (*p* = 0.04). We saw differences in “velocity of the team’ s movement” and “information gathering” but these were not significant.

According to the evaluation criteria and the cut-off values (Figs. 1, 2 and 3) of each item developed by Hagemann et al., the topics “interconnectedness: departments”, “interconnectedness: information flow”, “dynamics”, “polytely”, “velocity of the team’s movement”, “velocity of system changes”, “hierarchy” and “hierarchy: leadership”, “shared task mental model”, “shared team mental model” and all aspects of the scale “adaptive behaviors” should be implemented as focal points into CRM trainings (Table 1). Practical examples for the conceptualization of items and item groups are depicted in Table 1 for an understanding of the underlying concepts and to help to transfer the items into the daily work.

### System size

Overall, 97 participants gave information about the system size in their work environment. The system size is indicated as 2‑30 people (median 4.0, IQR (interquartile range) (3.0)). Teams in anesthesia were specified as 2–11 people (median 3.0, IQR 2.0), in intensive care medicine as 3–30 (median 7.0, IQR 20).

Free text responses indicated that team size can also change depending on the care required and clinical situation. In intensive care units (ICU), respondents indicated that an entire “shift” can be seen as a team. On the other hand, anesthesia participants indicated that teams almost always include two people, with only critical emergency situations involving more people.

## Discussion

The aim of the study was to investigate the work environment of German health care professionals. CRM uses standardized communication channels and tools to improve the efficiency and safety of patient care workflows. The employees with their CRM play an important role to compensate for unpredictable systemic deficits. Therefore, CRM should be trained. The goal of most training simulations is to improve CRM, not to identify errors in the processes of the ICU or the OR [14]. The results should be integrated into crew resource management courses in order to reflect the challenges faced in the work environment of teams as realistically as possible.

Buzzwords for conducting simulations and designing scenarios are given [4]. Due to the medical societies and the significant potential threat in corresponding emergency situations, technical contents such as airway management, management of malignant hyperthermia, bleeding management or a LAST are elementary components in most courses [25]. However, the specifics of the courses may vary.

TAKAI can broaden our perspective by describing the contexts in which the simulation scenarios take place. This aids us by providing options for course design, thereby making the simulations more realistic and applicable to the course contents. The existing scenarios could be evaluated with regard to the TAKAI dimensions. Subsequently, studies on the effectiveness of the revised scenarios can be conducted.

### Demographic data

The participants of this study were recruited from nine hospitals across Germany. The participants’ hospitals represented all levels of hospital care provided in Germany, and a broad distribution across the different age categories as well as the professional experience of the participants. The results are therefore not limited to individual levels but are most likely comparable.

We received responses from all professional groups involved in regular patient care. Participants from both nursing and medical services are involved in CRM training and play an important role in providing optimal patient care.

As previously described, the teams of different disciplines probably work in comparable work environments (anesthesia versus intensive care) [10, 19]—TAKAI is a way to evaluate a work environment independent of disciplines [19, 21].

### Experience with human factors, crew resource management and simulation-based training

Our data show that there tends to be subjectively less experience in the ICU we surveyed than in the anesthesia departments. In fact, despite the widespread use of simulation training as an established training concept, there are still staff members without experience in this type of training. As described above, age and work experience have an influence on the experience in simulation training. Staff with an age of 40–49 years or a work experience of 10–15 years have significantly higher experience with simulation training. For the development of a training program, it might be essential to actively integrate younger and “older” staff members to achieve an even distribution of CRM knowledge and skills.

Despite this, the importance of non-technical skills in the context of patient safety has been recognized for over 20 years [23], a significant proportion of the professionals have no to moderate experience in human factors and/or CRM. This result correlates with a recent survey conducted in our working group [10]. This lack of experience could be the result of the institutions educational and unit framework, lack of compulsory training, high costs and personnel shortage. The work context is not affected by simulation training experience, but a high potential need for simulation training in the environment studied is evident.

### Reliability of the scales

The reliability of the scales is acceptable and matches the validation data of TAKAI. As described by Hagemann et al., the items have a high correspondence with established CRM training concepts and therefore were not deleted from the questionnaire to improve the reliability [19]. We therefore used the survey data for further evaluation.

### System size

As described previously, respondent professional groups rate the system size of their work environment differently by specialty. ICU teams tend to see themselves more as one large team (complete shift of an ICU), while anesthesia teams tend to see themselves as smaller groups of 2–3 people. For the purpose of this thesis, we suggest that the critical care training should take place in a fully staffed ICU. In contrast, the smaller anesthesia teams may be able to be combined from different work areas or hospitals into one course since the “team” unit is smaller. This approach whether interdepartmental or intradepartmental delivery of simulation training, has a greater impact on the effect of simulation training and should be further explored.

Furthermore, the training design could take into account that ICU scenarios have more interfaces with the entire team (delegation of arterial blood gas, ABG, use of additional resources). It may also be useful to run two scenarios in parallel to reflect prioritization of tasks, resource allocation and task management.

As already described, an indiscriminate adoption of CRM training content from aviation is not reasonable [16]. The work contexts are so different that a differentiated consideration of the individual focal points is necessary. For example, a pilot always exposes himself to danger during an incident in flight, so dealing with personal threat can be an important aspect of training. This is not usually the case in medicine.

Other aspects such as a “shared mental model” must definitely be included in simulation training, since in medicine there is no linear cause-effect principle in which checklists can help. Situations and patients are complex and react differently to incidents and therapeutic measures [7, 31]. A shared mental model amongst attending persons is an important factor for the success of the therapy [9]. A digital aid to improve the shared mental model of teams and to deal with complex situations was introduced as the “German digital cognitive aid for crisis management in anesthesiology” (known as eGENA) by the German Society of Anesthesiology and Intensive Care Medicine [9, 26].

### Differences in the scales of TAKAI between the work environments and concretization of crew resource management training

The impact scenario and training design have on the effect of simulation training are not always the same [11]. TAKAI can be an additional way to better adapt training to the focus group by analyzing the teamwork context. We therefore hypothesized that the teamwork context differs in anesthesia and intensive care teams.

In the groups surveyed, we saw a significant difference in the item “familiarity of the work environment” and slight, although not significant differences in “velocity of the team’s movement” and “information gathering”. All three aspects are rated higher by anesthesia teams.

The item “familiarity with the work environment” gets a high rating from respondents in both work contexts. It can therefore be assumed that the employees in both areas have excellent knowledge of their work environment.

Nevertheless, a training environment should of course be designed in such a way that all participants can find their way around. This is especially necessary if no in situ training takes place [22]. On the other hand, this can also be seen as a need to conduct in situ training especially in ICU, as all different rooms (storage room and ABG laboratory) and equipment can be included. Distances to be covered and time required for item retrieval, preparation, and assembly should be represented realistically during training.

We interpret the point about information gathering to mean that anesthesia teams usually care for a well-prepared and informed patient. With optimal preparation, the patient’s previous illnesses and medical history are well known. Optimally, there are even current examination results before the start of anesthesia. Exceptions to this are emergency situations, for example in the trauma bay or resuscitation room, where this is not the case. However, these aspects can be taken into account when designing the training.

This is less often the case for teams in intensive care units where critically ill patients have been treated, histories have been taken and investigations are carried out prior to arrival—depending on the orientation of the unit, patients arrive on the unit and must first be examined and the medical history has to be taken. Information about the patient must first be collected. In addition, intensive care patients will often have a longer course of treatment and more complex clinical picture. Joint training can then lead to the exchange of mental models.

The changes in the patient during anesthesia are usually due to the operation, except in the case of adverse events. This proceeds in comprehensible steps.

In the ICU, the driver is often a disease with unplanned dynamics and a wide variety of unplanned changes (e.g. sepsis). Healthcare professionals have to get to the bottom of this first.

These differences can be incorporated into training sessions, allowing a simulation course to be tailored to the participant group. For example, anesthesia teams may need a file with important patient information, whereas ICU teams are more likely to need equipment provided to perform comprehensive diagnostics (sonography, blood gas analysis, and more).

We also saw a discrete difference in the velocity of the team’s movement. Here, according to Hagemann et al., an integration into a CRM training is reasonable (3.5 and more points) [19] but in relation to a commercial airliner or even a fighter jet, this might be critical to evaluate.

Nevertheless, it may make sense to develop a training scenario in which the team is in motion while caring for a patient—an example would be the transfer of a patient for an emergency cesarean section to the operating room [12] or a transport of a patient to a decentralized diagnostic unit [30].

**Fig. 4:**
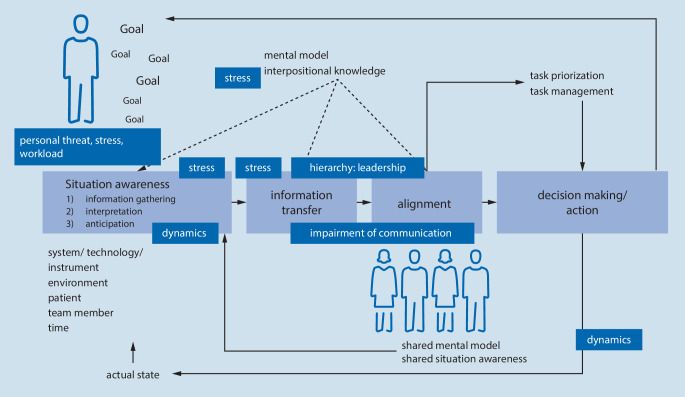
“Duisburg CRM model” [20, 29]. Shown is the process of “situation awareness” through communication with the team and decision making/implementation of actions. In addition, important aspects from the TAKAI analysis are overlaid at the corresponding points of the work process. The model shows a control loop of a team collaboration and allows the identification of classical non-technical skills and the matching items of TAKAI

In order to apply the aspects of TAKAI to CRM training and use them for debriefing purposes, Hagemann et al. use the “Duisburg CRM Model” ([19, 20, 29]; Fig. 4). In this model, the items of TAKAI are brought together with the known categories of non-technical skills [15, 18] and thus form a good overview of the interaction of teams in specific work areas. The model not only allows emphasis on certain contents of CRM training but also to incorporate variation of teaching methods into the training as needed (such as a role reversal of physician and nurse to experience different mental models to follow-up in the debriefing).

Overall, we see many similarities between the surveyed work contexts, but also the differences described. Therefore, the same training for all occupational fields does not seem reasonable, especially under the impression that not every aspect of CRM can be accommodated in every simulation course and therefore has to be selected.

In this study, we have described the working context of two HRT using TAKAI. In the future, we will have to adapt scenarios and training concepts accordingly and then test whether the presence and the learning effect on the participants is increased or the transfer of what has been learned into daily practice is more successful. TAKAI can help by allowing scenarios to be evaluated and improved after they have been created, also in terms of the target group’s working context.

### Limitations

This study was conducted during the period of the second wave of the SARS-CoV‑2 pandemic. At that time, intensive care medicine in Germany was extremely burdened by very high patient caseloads.

The low (but still acceptable) number of participants from intensive care medicine can be adequately explained by this. According to the validation of TAKAI, the number of participants from ICU was sufficiently large. In addition, it was found that professionals with different prior experience in CRM and simulation participated. Also, it was found that professionals with different prior experience in CRM and simulation participated.

Although a multicenter approach was chosen for this study, a different picture may still emerge across the entire cohort of all departments in Germany. Further analyses of work environments are required.

The survey did not ask about the participants’ medical specialty; however, since not all intensive care units are structured in the same way across specialities (for example, surgical, internal medicine, or interdisciplinary ICUs). The individual orientation of the ICU may have an influence on the ratings.

## Conclusion

The TAKAI scales meet the quality criteria (Cronbach’s alpha > 0.6) of the professional groups of anesthesia and intensive care professionals in German hospitals and can be used for analysis of the teamwork environment. The results indicate many similarities between the work contexts surveyed, but also slight differences. TAKAI can be an additional method to design appropriate simulation training for HRT from anesthesia and intensive care medicine as there does not seems to be a one-size-fits-all simulation solution. For a special focus on the needs of a particular work context—after introduction to the methodology—the easy to perform TAKAI analysis in the needs analysis step is worthwhile.

## Supplementary Information

Additional figures

Demographic data

Questionnaire

## Abbreviations

CRM: Crew resource management
HRT: High responsibility teams
ICU: Intensive care unit
LAST: Local anesthetic systemic toxicity
MH: Malignant hyperthermia
OR: Operating room
TAKAI: *Team-Arbeit-Kontext-Analyse Inventar *(Teamwork context analysis inventory)

## Declarations

This study was reviewed and approved by the Ethics Committee of the Medical School Hannover under the processing number 9445_BO_K_2020 (Chairperson: Professor S. Engeli). All participants consented to the collection of questionnaire data. Consent for publication: not applicable.
