# Supplementary material for: Further development of crew resource management training: Needs assessment by means of teamwork-context analysis in anesthesia and intensive care teams
Source: Anaesthesiologie. 2022 Jul 8;71(Suppl 2):180–9. doi: 10.1007/s00101-022-01170-3 (PMC9266080; doi:10.1007/s00101-022-01170-3)

Supplementary material to the article "Further development of crew resource management training - needs assessment by means of team-work-context-analysis in teams of anaesthesia and intensive care" by Eismann H, Breuer G, Flentje M (2022) in *Die Anaesthesiologie.*

Article and supplementary material are available at www.springermedizin.de. Please enter the article title in the search there.

Additional Figures

Experience in human factors and/or crew resource management on a scale from "no experience" to "very much experience".

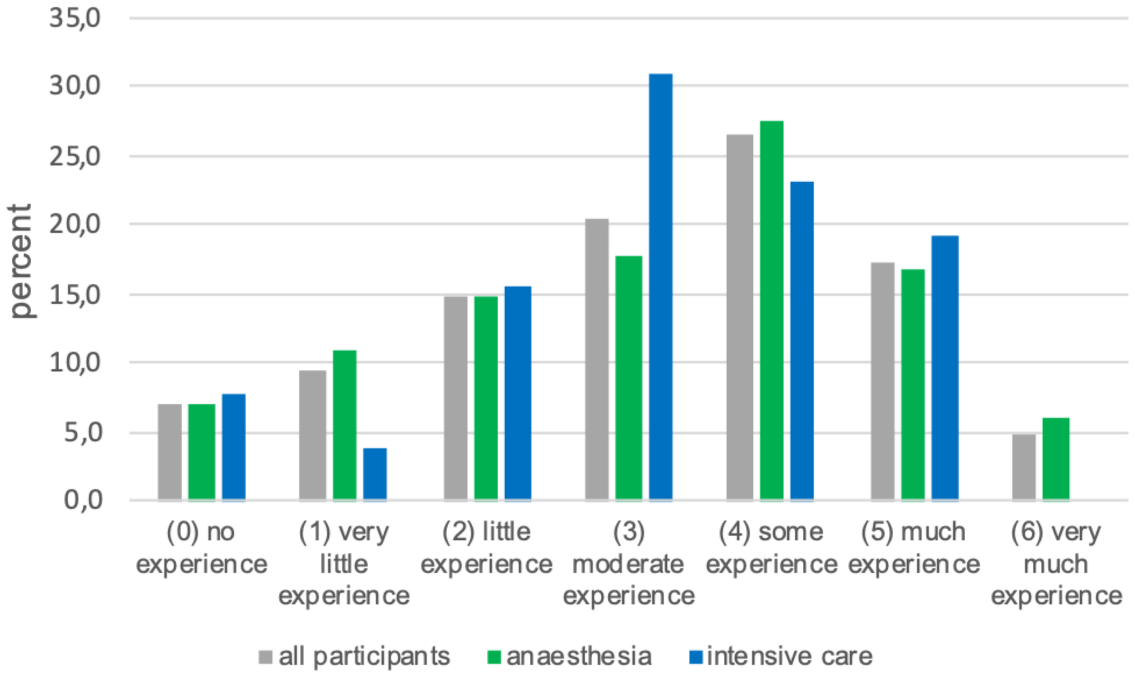

Experience in the methodology "simulation as team training" on a scale from "no experience" to "very much experience".

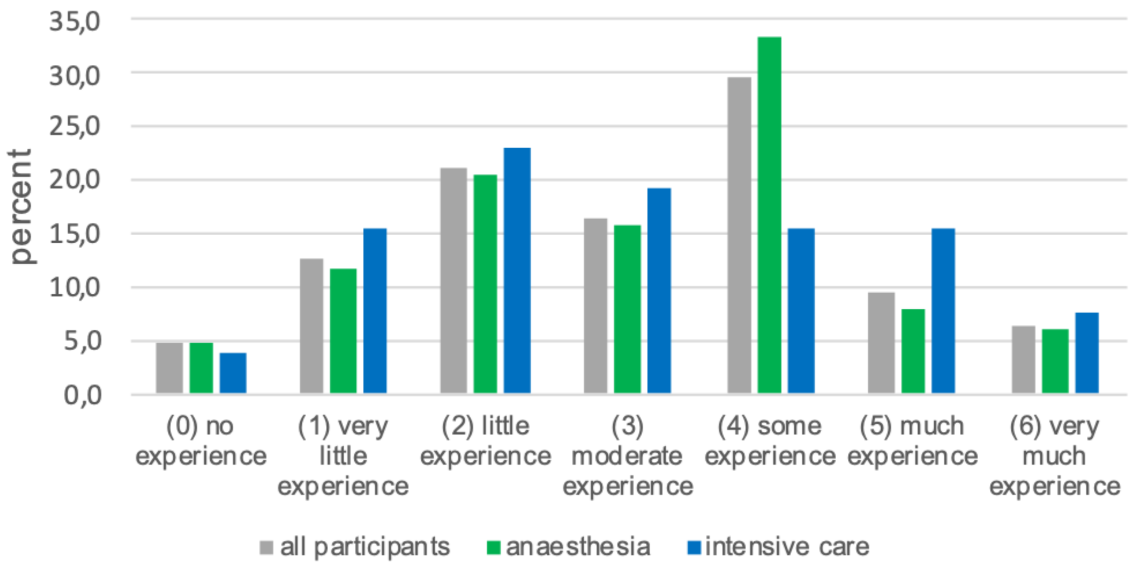

Supplement: Supplementary file 1 — Additional figures [file 101_2022_1170_MOESM1_ESM.pdf]
