# Supplementary material for: Further development of crew resource management training: Needs assessment by means of teamwork-context analysis in anesthesia and intensive care teams
Source: Anaesthesiologie. 2022 Jul 8;71(Suppl 2):180–9. doi: 10.1007/s00101-022-01170-3 (PMC9266080; doi:10.1007/s00101-022-01170-3)
Supplement: Supplementary file 2 — Demographic data [file 101_2022_1170_MOESM2_ESM.pdf]

Supplementary material to the article "Further development of crew resource management training - needs assessment by means of team-work-context-analysis in teams of anaesthesia and intensive care" by Eismann H, Breuer G, Flentje M (2022) in *Die Anaesthesiologie.*

Article and supplementary material are available at www.springermedizin.de. Please enter the article title in the search there.

Demographic data of the participants.

		**frequenc**	**percentage**
**sex**	male	57	44.5
	female	69	53.9
	non-binary	2	1.6
**Age [years]**	20-29	21	16.4
	30- 39	41	32.0
	40-49	30	23.5
	50-59	26	20.3
	60-69	10	7.8
**work experience [years]**	< 2	20	15.6
	2-5	15	11.7
	5-10	23	18
	10-15	15	11.7
	> 15	55	43
**level of hospital care**	First-level hospital	12	9.5
	Second-level hospital	7	5.6
	Third-level hospital	49	38.9
	university hospital	58	46.0
	not answered	2	
**profession**	nurse (anaesthesia)	7	5.5
	nurse (intensive care medicine)	5	3.9
	specialised nurse (anaesthesia)	13	10.2
	specialised nurse (intensive care medicine)	3	2.3
	anaesthesia technician	3	2.3
	residents (anaesthesia)	29	22.7
	residents (intensive care medicine)	6	4.7
	consultant (anaesthesia)	50	39.1
	consultant (intensive care medicine)	12	9.4
**work environment**	anaesthesiology	102	79.8
	intensive care medicine	26	20.3
