# Supplementary material for: Further development of crew resource management training: Needs assessment by means of teamwork-context analysis in anesthesia and intensive care teams
Source: Anaesthesiologie. 2022 Jul 8;71(Suppl 2):180–9. doi: 10.1007/s00101-022-01170-3 (PMC9266080; doi:10.1007/s00101-022-01170-3)
Supplement: Supplementary file 3 — Questionnaire [file 101_2022_1170_MOESM3_ESM.pdf]

Supplementary material to the article "Further development of crew resource management training - needs assessment by means of team-work-context-analysis in teams of anaesthesia and intensive care" by Eismann H, Breuer G, Flentje M (2022) in Die Anaesthesiologie.

Article and supplementary material are available at www.springermedizin.de. Please enter the article title in the search there.

German language version of the TAKAI-questionnaire

Question	Answers
**Welches Geschlecht haben Sie?**	[weiblich, männlich, divers]
**Wie alt sind Sie?**	[<19 Jahre, 20-29 Jahre, 30-39 Jahre, 40-49 Jahre, 50-59 Jahre, 60-69 Jahre]
**Bitte geben Sie Ihre Berufserfahrung in der Anästhesie und Intensivmedizin an.**	[<2 Jahre, 2-5 Jahre, 5-10 Jahre, 10-15 Jahre, >15 Jahre]
**Welcher Berufsgruppe gehören Sie an?**	[Pflegekraft in der Anästhesie, Pflegekraft in der Intensivmedizin, Fachpflegekraft in der Anästhesie, Fachpflegekraft in der der Intensivmedizin, Anästhesietechnischer Assistant/ Assistentin, Arzt/Ärztin in Weiterbildung zur Zeit in der Anästhesie, Arzt/Ärztin in Weiterbildung zur Zeit in der Intensivmedizin, Fach-/ Oberarzt zur Zeit in der Anästhesie, Fach-/ Oberarzt zur Zeit in der Intensivmedizin]

**Wo sind Sie beschäftigt?**	[Praxis, Krankenhaus der Grund- und Regelversorgung, Krankenhaus der Schwerpunktversorgung, Krankenhaus der Maximalversorgung, Universitätsklinik]
**Wie schätzen Sie Ihre Erfahrung in den Bereichen „Human Factors“ und/ oder „CRM – Crew Resource Management“ ein?**	[0 (keine Erfahrung) - 6 (sehr viel Erfahrung)]
**Wie schätzen Sie Ihre Erfahrung in der Methodik „Simulation als Teamtraining“ ein?**	[0 (keine Erfahrung) - 6 (sehr viel Erfahrung)]
**In unserem Arbeitsfeld können Informationen, die zur Lösung einer Problemsituation benötigt werden, nicht besorgt werden.**	[0 (trifft nie zu) - 6 (trifft immer zu)]
**Wir haben in unserer Arbeit alle Daten und Fakten, die uns helfen schnell und richtig zu entscheiden.**	[0 (trifft nie zu) - 6 (trifft immer zu)]
**In Problemsituationen sind hilfreiche Informationen nicht bekannt.**	[0 (trifft nie zu) - 6 (trifft immer zu)]
**Informationen, auf die wir uns bei Entscheidungen verlassen, stellen sich im Nachhinein als falsch heraus.**	[0 (trifft nie zu) - 6 (trifft immer zu)]
**Für eine erfolgreiche Erfüllung der Aufgabe ist die Zusammenarbeit von mehreren Abteilungen bei uns nötig.**	[0 (trifft nie zu) - 6 (trifft immer zu)]

**Wenn in einer Abteilung Informationen oder Materialien fehlen, hat dies auch Konsequenzen für andere Abteilungen.**	[0 (trifft nie zu) - 6 (trifft immer zu)]
**In Problemsituationen werden Informationen zwischen verschiedenen Positionsinhabern weitergegeben.**	[0 (trifft nie zu) - 6 (trifft immer zu)]
**Wenn in einer Abteilung Informationen oder Materialien fehlen, hat dies auch Konsequenzen für andere Abteilungen.**	[0 (trifft nie zu) - 6 (trifft immer zu)]
**In unserer Arbeit verändern sich Situationen auch ohne unser Handeln.**	[0 (trifft nie zu) - 6 (trifft immer zu)]
**Die Zeit ist ein sehr wichtiger Faktor in unserem Arbeitsfeld.**	[0 (trifft nie zu) - 6 (trifft immer zu)]
**Entscheidungen müssen unter zeitlichen Begrenzungen getroffen werden.**	[0 (trifft nie zu) - 6 (trifft immer zu)]
**In unserem Arbeitsfeld können wir unsere Aufgaben für eine gewisse Zeit auf die Seite legen und zu einem späteren Zeitpunkt daran weiterarbeiten ohne schlimme Auswirkungen befürchten zu müssen.**	[0 (trifft nie zu) - 6 (trifft immer zu)]
**In unseren Handlungen zur Lösung von Problemen müssen wir gleichzeitig mehrere Ziele (Bspw. Sicherheit & Schnelligkeit) berücksichtigen.**	[0 (trifft nie zu) - 6 (trifft immer zu)]
**Um unsere Aufgaben erfolgreich zu erfüllen, müssen wir mehrere Ziele gleichzeitig beachten.**	[0 (trifft nie zu) - 6 (trifft immer zu)]

**In unserer Arbeit stehen Ziele, die zur Lösung einer Problemsituation erreicht werden müssen, miteinander in Konkurrenz.**	[0 (trifft nie zu) - 6 (trifft immer zu)]
**Unsere Arbeit ist durch ein Abwägen von Vorund Nachteilen für die Erreichung von verschiedenen Zielen gekennzeichnet.**	[0 (trifft nie zu) - 6 (trifft immer zu)]
**In der Arbeit haben unsere Handlungen direkte Konsequenzen, so dass wir sofort wissen, was wir bewirkt haben.**	[0 (trifft nie zu) - 6 (trifft immer zu)]
**Die Ergebnisse unserer Handlungen sind uns direkt danach bekannt.**	[0 (trifft nie zu) - 6 (trifft immer zu)]
**Auf unsere Handlungen erhalten wir sofort eine Rückmeldung.**	[0 (trifft nie zu) - 6 (trifft immer zu)]
**Bewegen Sie sich oder das Team während der Arbeit für eine erfolgreiche Erfüllung Ihrer Aufgaben?**	[0 (trifft nie zu) - 6 (trifft immer zu)]
**Geben Sie bitte von 0 (wie bspw. ein Fussgänger) bis 6 (wie bspw. ein Kampfflugzeug) an, wie schnell Sie sich bewegen.**	0 (Fussgänger) - 6 (Kampfflugzeug)
**Bitte geben Sie an, aus wie vielen Personen, inklusive Ihnen selbst, Ihr Team besteht.**	[0 (trifft nie zu) - 6 (trifft immer zu)]
**In unserer Arbeit verändern sich die Situationen, auf die wir Einfluss nehmen müssen, sehr schnell.**	[0 (trifft nie zu) - 6 (trifft immer zu)]

**n unserem Arbeitsfeld müssen wir Entscheidungen sehr schnell treffen.**	[0 (trifft nie zu) - 6 (trifft immer zu)]
**In unserer Arbeit stellt sich eine Veränderung in den Bedingungen der Situation von jetzt auf gleich ein.**	[0 (trifft nie zu) - 6 (trifft immer zu)]
**Die Zeit ist ein sehr wichtiger Faktor für Entscheidungen in unserer Arbeit.**	[0 (trifft nie zu) - 6 (trifft immer zu)]
**In unserer Arbeit hat Hierarchie einen grossen Einfluss auf unser Verhalten.**	[0 (trifft nie zu) - 6 (trifft immer zu)]
**In unserer Arbeit wird allen Anweisungen von Vorgesetzten Folge geleistet, komme was wolle.**	[0 (trifft nie zu) - 6 (trifft immer zu)]
**Auf die Einhaltung des Dienstweges wird in unserer Arbeit grossen Wert gelegt.**	[0 (trifft nie zu) - 6 (trifft immer zu)]
**Während der Arbeit werden Anweisungen von oben hinterfragt, wenn das für eine Lösung der Situation bedeutend ist.**	[0 (trifft nie zu) - 6 (trifft immer zu)]
**In der Arbeit werden Informationen und Anmerkungen von Rangniedrigeren in die Lösungsfindung mit einbezogen.**	[0 (trifft nie zu) - 6 (trifft immer zu)]
**In unserer Arbeit sind wir extremer Hitze ausgesetzt.**	[0 (trifft nie zu) - 6 (trifft immer zu)]
**Während der Arbeit können wir nichts oder nicht richtig sehen.**	[0 (trifft nie zu) - 6 (trifft immer zu)]
**In unserer Arbeit sind wir extremer Kälte ausgesetzt.**	[0 (trifft nie zu) - 6 (trifft immer zu)]

**Während der Arbeit müssen wir bei Dunkelheit und schlechtem Licht arbeiten.**	[0 (trifft nie zu) - 6 (trifft immer zu)]
**Arbeitsumgebungen wie Gewässer oder heftiger Regen haben einen großen Einfluss auf die Erfüllung unserer Aufgabe.**	[0 (trifft nie zu) - 6 (trifft immer zu)]
**In unserer Arbeit sind wir äußeren Bedingungen wir Sturm ausgesetzt.**	[0 (trifft nie zu) - 6 (trifft immer zu)]
**Während der Arbeit können wir mit unseren Kollegen ungehindert kommunizieren.**	[0 (trifft nie zu) - 6 (trifft immer zu)]
**In Problemsituationen ist der Austausch von Informationen zwischen den handelnden Personen völlig barrierefrei.**	[0 (trifft nie zu) - 6 (trifft immer zu)]
**Zur Lösung einer Problemsituation können wir nur so miteinander kommunizieren, dass eine vollständige Informationsübermittlung nicht gewährleistet ist.**	[0 (trifft nie zu) - 6 (trifft immer zu)]
**Für die Erfüllung unserer Aufgaben arbeiten wir auch an Plätzen, an denen wir noch nie zuvor gewesen sind.**	[0 (trifft nie zu) - 6 (trifft immer zu)]
**In Problemsituationen handeln wir in einer uns vertrauten Arbeitsumgebung.**	[0 (trifft nie zu) - 6 (trifft immer zu)]
**Während der Arbeit wechseln wir in eine Arbeitsumgebung, die uns unbekannt ist.**	[0 (trifft nie zu) - 6 (trifft immer zu)]
**Während unserer Arbeit handeln wir an Orten, die uns vertraut sind.**	[0 (trifft nie zu) - 6 (trifft immer zu)]

**In unserer Arbeit steht die erfolgreiche Bewältigung einer Problemsituation in direktem Zusammenhang zu unserem eigenen Überleben.**	[0 (trifft nie zu) - 6 (trifft immer zu)]
**Fehler in unserer Arbeit haben Konsequenzen für unser Überleben.**	[0 (trifft nie zu) - 6 (trifft immer zu)]
**Es ist wichtig die berufliche Umwelt und Technologie zu verstehen und zu wissen, wie die Teamkollegen darin agieren.**	[0 (trifft nie zu) - 6 (trifft immer zu)]
**Es ist von großer Bedeutung eine volle Kenntnis der beruflichen Ausrüstung zu haben sowie das Wissen der Kollegen darüber zu erfahren.**	[0 (trifft nie zu) - 6 (trifft immer zu)]
**Innerhalb des Teams ist es wichtig ein gemeinsames Verständnis über die zu erledigenden Aufgaben zu haben.**	[0 (trifft nie zu) - 6 (trifft immer zu)]
**Es ist zentral eine gemeinsame Auffassung im Team über die Strategien und Vorgehensweisen zur Erfüllung der Aufgaben zu haben.**	[0 (trifft nie zu) - 6 (trifft immer zu)]
**Im Team ist es wichtig zu wissen, welche Rollen und Verantwortlichkeiten die einzelnen Mitglieder haben.**	[0 (trifft nie zu) - 6 (trifft immer zu)]
**Es ist wichtig zu wissen, wer im Team mit wem zusammenarbeitet und aufeinander angewiesen ist.**	[0 (trifft nie zu) - 6 (trifft immer zu)]
**Das Wissen und die Fähigkeiten der Teamkollegen zu kennen ist von grosser Bedeutung.**	[0 (trifft nie zu) - 6 (trifft immer zu)]

**Es ist wichtig die Stärken und Schwächen sowie Einstellungen der Teamkollegen zu kennen.**	[0 (trifft nie zu) - 6 (trifft immer zu)]
**Es ist wichtig Informationen aus der Umwelt über die Situation und die Aufgabe zu sammeln, um bewerten zu können, wann gehandelt werden muss.**	[0 (trifft nie zu) - 6 (trifft immer zu)]
**Die erhobenen Informationen müssen in den Arbeits-/Situationskontext eingebettet und darin interpretiert werden, um zu verstehen was sie bedeuten.**	[0 (trifft nie zu) - 6 (trifft immer zu)]
**Mit den gesammelten Informationen sollte man zukünftige Situationszustände oder Probleme vorhersagen.**	[0 (trifft nie zu) - 6 (trifft immer zu)]
**Anstehende Aufgaben zu planen und zu priorisieren ist sehr wichtig.**	[0 (trifft nie zu) - 6 (trifft immer zu)]
**Es hilft uns Aufgaben während nicht belastenden Situationen zu priorisieren, da wir dann dieses Schema auf kritische Situationen übertragen und abarbeiten können.**	[0 (trifft nie zu) - 6 (trifft immer zu)]
**n kritischen Situationen tendieren wir dazu Priorisierungen von Aufgaben rückgängig zu machen und neu vorzunehmen.**	[0 (trifft nie zu) - 6 (trifft immer zu)]
**Eine effektive Aufgabenverteilung über alle Teammitglieder ist sehr wichtig.**	[0 (trifft nie zu) - 6 (trifft immer zu)]

**In unserer Arbeit ändern wir wenn nötig die Aufgabenverteilung, so dass die Arbeitsbelastung im Team gleichverteilt bleibt.**	[0 (trifft nie zu) - 6 (trifft immer zu)]
